# Nutrients and chemical composition of *Desplatsia dewevrei*

**DOI:** 10.1002/fsn3.1019

**Published:** 2019-04-09

**Authors:** Oghale Ovuakporie‐Uvo, MacDonald Idu, Ehimwenma Sheena Omoregie

**Affiliations:** ^1^ Department of Biological and Chemical Sciences, Faculty of Natural and Applied Sciences Michael and Cecilia Ibru University Ughelli Delta State Nigeria; ^2^ Phytomedicine Research Group, Department of Plant Biology and Biotechnology University of Benin Benin City Edo State Nigeria; ^3^ Department of Biochemistry University of Benin Benin City Edo State Nigeria

**Keywords:** anti‐nutritional, *Desplatsia dewevrei*, nutraceutical, nutritional

## Abstract

Several plants species have served as food for man because of their nutritional values. However, there is a lack of data on the nutritional benefits of *Desplatsia dewevrei*. Hence, this study was aimed at investigating the nutrients and chemical composition of *D. dewevrei* leaves and fruits. Vitamins, amino acid profile, and mineral composition of *D. dewevrei* were investigated. Minerals found present include copper, magnesium, calcium, and potassium. Vitamins B, C, and K up to the daily standard required intake levels according to World Health Organization standards were discovered in *D. dewevrei*. The protein score from the amino acid composition was 100%. Phytate and oxalate, which are non‐nutritional components, were found present in *D. dewevrei;* however, in values far below the daily intake required limit by the WHO standard. In conclusion, *D. dewevrei* from this research finding has convincing qualities of a reliable nutraceutical raw material, which can be properly finished and incorporated into the human diet to harness its vitality.

## 1 INTRODUCTION

In recent past years, an increasing number of plant‐derived dietary supplements have become available in supermarkets and health food shops. These supplements, which elicit diverse mild pharmacological effects, are collectively referred to as “Nutraceutical.” The term “Nutraceutical” used to describe “a medicinally or nutritionally functional food was coined in 1989 by Stephen De Felice, founder and chairman of the Foundation for Innovation in Medicine” (Jack, 1995; Mannion, 1998). Palatable seeds are essential sources of nutrients and energy particularly among the resource‐poor people where protein‐energy undernourishment has insistently hindered peak growth and development (Escudero et al., 2006; Perumal, Klaus, & Harinder, 2001). Food crops on which most human nutrition is based depend on photosynthesis while ethnomedicines, drugs, and essential oils are made from plant secondary products of metabolism such as the alkaloids, terpenoids, and flavonoids (Alaribe, 2008).

*Desplatsia dewevrei* is a scarce West African plant species commonly referred to as “bush okra.” Indigenous people use *D. dewevrei* chiefly in making delicacies; however, a few herbalists use the plant parts as medicine (Ovuakporie‐Uvo & Idu, 2017). This study was aimed at investigating the nutrients and chemical composition of *D. dewevrei*.

## 2 MATERIALS AND METHODS

Nutritional studies were carried out following methods of A.O.A.C. (2016). Some of them are as follows:

### 2.1 Determination of ash content

A preheated and cooled crucible was weighed. The sample was charred on a Bunsen flame inside a fume cupboard. The charred sample was placed in a muffle furnace set at 550⁰C for 2 hr until a white or light gray ash is obtained. The sample was removed, cooled in desiccators, and weighed.

$$\text{Ash content}=\frac{W_{3}-W_{1}}{W_{2}-W_{1}}\times 100$$

Where *W*_1_ = weight of empty crucible.

*W*_2_ = weight of crucible + weight of sample.

*W*_3_ = weight of crucible + weight of the sample after ashing.

### 2.2 Determination of crude fiber

The sample was oven dried at 105⁰C. Powdered dried sample (2 g) was placed in a 500‐ml beaker, and 200 ml of boiling 1.25% H_2_SO_4_ was added. The beaker was placed on a hot plate and boiled for 3 min with occasional rotation of the beaker. The beaker was cooled and filtered by suction through a Buchner funnel. The beaker was rinsed with two 50 ml positions of boiling water. The residue was carefully transferred into a beaker, and 200 ml of 1.25% NaOH was added. It was boiled for 30 min, cooled and filtered, and washed twice with 50 ml boiling water.

Finally, the sample was washed with 25 ml 95% alcohol. The residue was oven dried for 2 hr at 130⁰C and cooled in a desiccator and weighed. The sample was heated for 30 min at 600⁰C and cooled in a desiccator and weighed.

$$\text{Crude fiber =}\;\frac{\text{Weight of residue}}{\text{Weight of sample}}\times 100$$

### 2.3 Determination of moisture content

The crucibles were washed and dried in an oven at 100⁰C for 1 hr. The weight was noted as W_1_. Two grams (2 g) of each sample was separately weighed into the crucibles, and their weights were taken and noted down as (W_2_) before and during drying at 100⁰C to constant weight (W_3_).

$$\text{Moisture content}=\frac{W_{2}-W_{3}}{W}\times 100=\frac{\text{weight of moisture}}{\text{weight of sample}}\times 100$$

where.

*W*_1_ = weight of empty crucible.

*W*_2_ = weight of crucible and sample before drying.

*W*_3_ = weight of sample after drying to a constant weight.

### 2.4 Determination of carbohydrate

Carbohydrate was estimated as the remainder after accounting for all, crude fiber, protein, and fats. Total carbohydrate contents = 100 − (% moisture + % ash + % protein + % fat).

### 2.5 Determination of phytate

The phytate content were determined using the method of Young and Greaves (1940) as adopted by Lucas and Markakes (1975).

Two hundred milligram (0.2 g) of the sample was weighed into two 250‐ml conical flasks. Each sample was soaked in 100 ml of 2% concentrated HCl for 3 hr. The sample was then filtered. Fifty (50) mls of each filtrate was laced in 250‐ml beaker, and 10 ml distilled water added to each sample. Ten (10) ml of 0.3% ammonium thiocyanate solution was added as an indicator and titrated with standard iron (III) chloride solution which contained 0.00195 g iron per 1 ml. Endpoint is observed to be yellow which persists for 5 min.

$$\text{\textbackslash{}\% Phytic acid}=\frac{\text{Titre value}\times 0.00195\times 1.19}{2}\times 100$$

### 2.6 Determination of oxalate

75 ml of 3M H_2_SO_4_ was added to 1g of the pulverized sample, and the solution was carefully stirred intermittently with a magnetic stirrer for 30 min thereafter filtered using Whatman No 1 filter paper. Twenty‐five (25 ml) of the filtrate was collected and titrated while hot against 0.1 N KMnO_4_ solution (0.05M KMnO_4_) until a faint pink color appeared that persisted for 30 s (Chinma & Igyor, 2007; Ihekoronye & Ngoddy, 1985).

1 ml KMnO4 = 2.2 mg Oxalate

Oxalate content = titer value × 2.2 mg.

### 2.7 Amino acid and vitamin composition

Amino acid and mineral analysis were carried out following modified A.O.A.C. (2006) method and Danka, Dobrina, and Kalin (2012). Water‐ and fat‐soluble vitamin determination was done following methods described by Dionex Corporation Annual Report (2010).

### 2.8 Mineral and elemental composition

Freshly prepared aqua regia solution was used in the analysis of the samples. For this, distilled water (1.2 L) was poured into a 2‐L volumetric flask. Then, 400 ml concentrated HCl was carefully added, followed with 133 ml of 70% nitric acid. The solution was made up to the mark with distilled water. Two (2) g of dry powder of *D. dewevrei* was placed in a crucible and ashed in a muffle furnace at 500°C for 2 hr after which it was allowed to cool in an oven, then removed and stored in a desiccator. The ashed material was transferred into a 50‐ml centrifuge tube by rinsing the crucible with 5 ml of distilled water. The crucible was further rinsed with 5 ml of the aqua regia about three times and poured into the tube to make a total of 20 ml. The sample was then vortexed for 10 min at 3,000 rpm which the supernatant was decanted into clean vials for macronutrients and micronutrients determination using flame atomic absorption spectrophotometer. The minerals analyzed included calcium, magnesium, potassium, sodium, and iron.

### 2.9 Statistical analysis

Data are stated as Mean ± Standard Error of Mean (*SEM*). Statistical analyses were carried out with one‐way analysis of variance (ANOVA). Multiple comparisons were done using Duncan multiple range tests (SPSS version 23), Tukey's multiple range tests (Graph pad prism 6), and Microsoft Excel software package. Significant levels were determined at *p* < 0.05.

## 3 RESULTS

**Figure 1:**
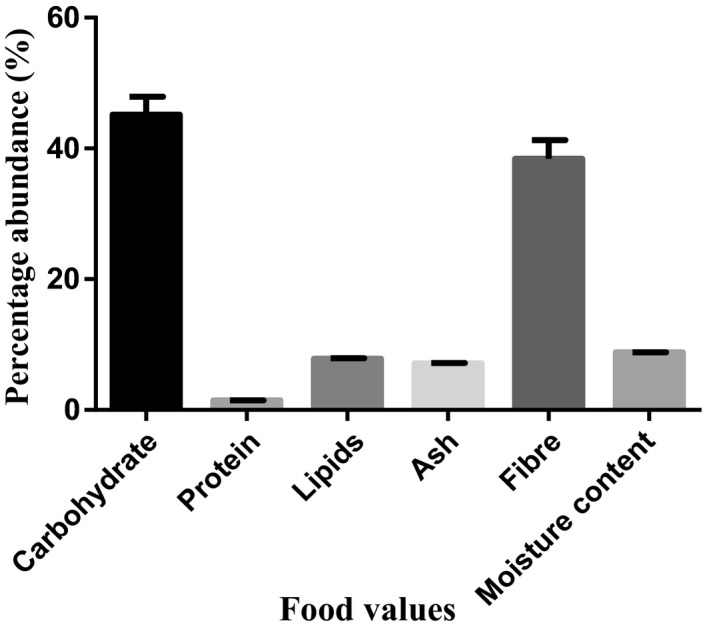
Proximate analysis of food value in *Desplatsia dewevrei* leaves. *n* = 3 determinations

**Figure 2:**
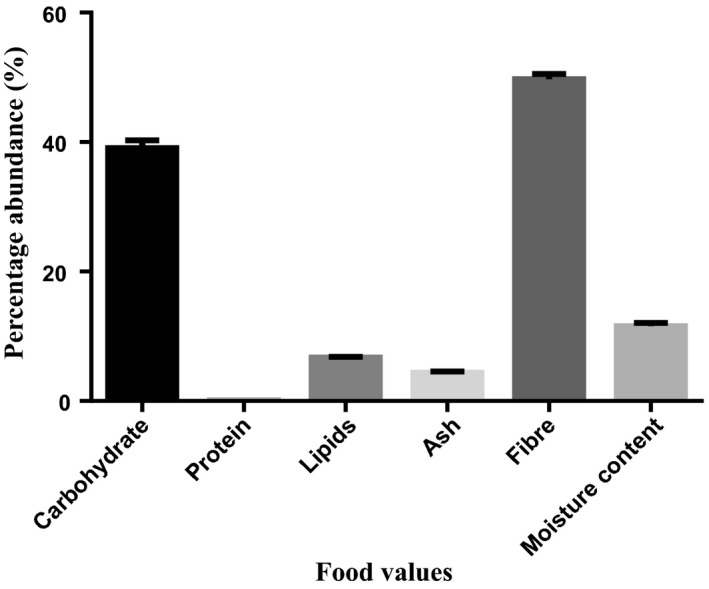
Proximate analysis of food value in *Desplatsia dewevrei* fruits. *n* = 3 determinations

The percentage compositions of nutritional values in *D. dewevrei* on dry weight basis show that the leaves and fruits of the plant are rich in carbohydrate and fiber. However, the leaves had more carbohydrate (45.16 ± 2.78) than the fruits (39.07 ± 0.87) while the fruits had more fiber content (49.71 ± 0.80) than the leaves (38.43 ± 2.85). Moisture was found to be more in the fruits (11.57 ± 0.36) than in the leaves (8.81 ± 0.01) of the plant as presented in Figures 1 and 2.

**Figure 3:**
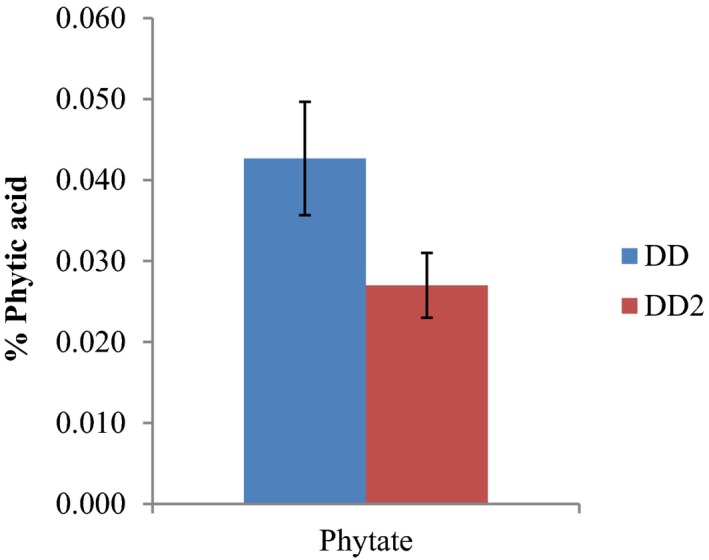
Percentage phytic acid Content of *Desplatsia dewevrei* leaves and fruits. Key: DD‐leaves; DD2‐fruits. *n* = 3 determinations

**Figure 4:**
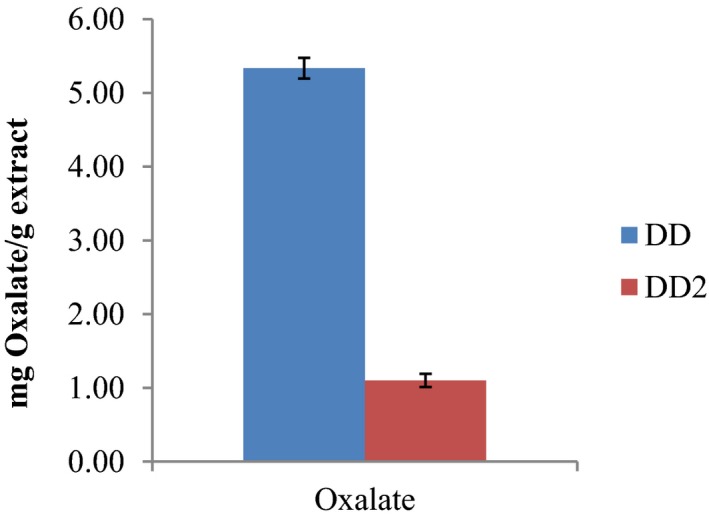
Oxalate Content of *Desplatsia dewevrei* leaves and fruits. Key: DD‐leaves; DD2‐fruits. *n* = 3 determinations

Phytate content of *D. dewevrei* was trace in the leaves (0.04 ± 0.01) and in the fruits (0.03 ± 0.00). The oxalate content was found to be more in the leaves (5.34 ± 0.14) than in the fruits (1.10 ± 0.09) as represented in Figures 3 and 4, respectively.

Mineral analysis of leaves and fruits revealed that the dried leaves contained a higher amount of copper (14.869 ppm), calcium (2.926 ppm), magnesium (5.426 ppm), and potassium (6.584 ppm) compared to the respective amounts of minerals and elements in the dried fruits (Table 1).

**Table 1:** Mineral composition of *Desplatsia dewevrei* leaves and fruits

Minerals	Leaves	Fruits
	Concentration (ppm)	Concentration (ppm)
Selenium	0.168 ± 0.01	0.032 ± 0.03
Chromium	0.028 ± 0.00	0.014 ± 0.01
Copper	14.869 ± 0.04	2.666 ± 0.00
Manganese	0.175 ± 0.01	0.038 ± 0.01
Iron	0.893 ± 0.02	0.145 ± 0.02
Sodium	0.286 ± 0.01	0.068 ± 0.02
Calcium	2.926 ± 0.00	1.187 ± 0.01
Magnesium	5.426 ± 0.01	1.664 ± 0.03
Potassium	6.584 ± 0.00	3.679 ± 0.01
Zinc	0.415 ± 0.03	0.136 ± 0.00

Note

*n* = 3 determinations.

The essential amino acids present in *D. dewevrei* were more in the leaves than fruits. Table 2 showed that glycine (4.426 g/ 100 g), alanine (4.181 g /100 g), serine (5.264 g/ 100 g), proline (3.051 g /100 g), aspartate (6.265 g /100 g), glutamate (17.556 g/ 100 g), tyrosine (2.352 g/ 100 g), and cytosine (2.029 g/ 100 g) were more quantifiable in the leaves of *D. dewevrei*. However, some nonessential amino acids, threonine (3.649 g/ 100 g), methionine (6.927 g/ 100 g), and histidine (1.949), were relatively more abundant in the fruits than the leaves.

**Table 2:** Essential and non‐essential amino acid composition of *Desplatsia dewevrei* leaves and fruits

Nonessential amino acids	Amount g/100g proteins		Essential Amino Acids	Amount g/100g proteins	
	Leaves	Fruits		Leaves	Fruits
Glycine	4.426	3.129	Valine	3.170	2.581
Alanine	4.181	2.680	Threonine	3.293	3.649
Serine	5.264	1.779	Isoleucine	4.459	1.800
Proline	3.051	1.839	Leucine	5.387	4.181
Aspartate	6.265	5.963	Lysine	3.307	3.201
Glutamate	17.556	8.815	Methionine	1.272	6.927
Tyrosine	2.352	2.612	Phenylalanine	7.451	3.249
Cystine	2.029	1.664	Histidine	1.605	1.949
			Arginine	8.154	1.822

**Figure 5:**
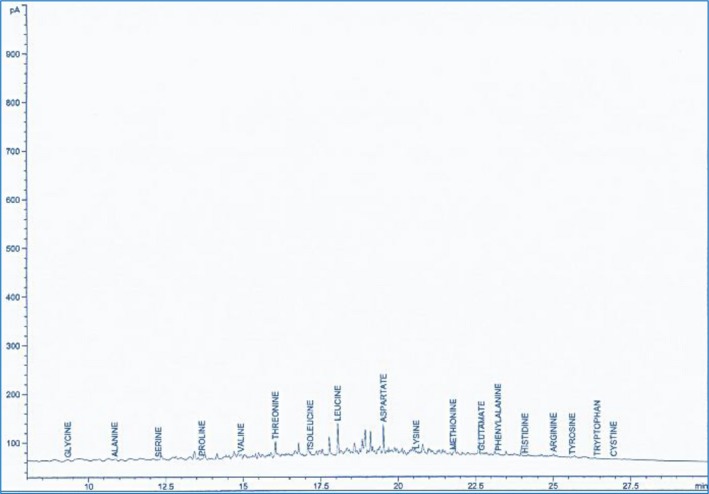
GC‐MS amino acid chromatogram of *Desplatsia dewevrei* methanol leaf extract

**Figure 6:**
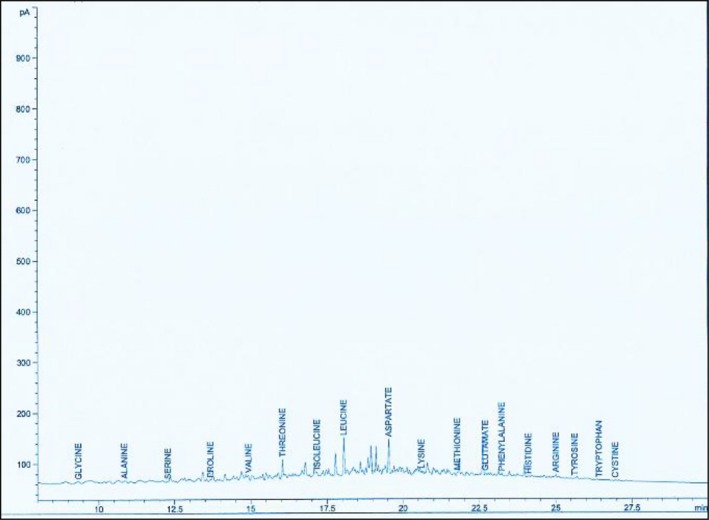
GC‐MS of amino acid chromatogram of *Desplatsia dewevrei* methanol fruit extract

The GC‐MS spectra of the methanol leaf and fruit extracts of *D. dewevrei,* showing the essential and nonessential amino acids, are presented in Figures 5 and 6.

Vitamin composition of *D. dewevrei* showed that vitamins A, B_1_, B_2_, B_3_, B_5_, B_6_, C, D, E, and K were more in the leaves than fruits. Conversely, vitamins B_12_ and B_9_ were more in the fruits than the leaves as represented in Table 3.

**Table 3:** Vitamin composition of *Desplatsia dewevrei* leaves and fruits

Total Vitamins	Amount g/100 g proteins	
	Leaves	Fruits
Vitamin B3	5.360	3.017
Vitamin B6	2.753	2.307
Vitamin C	53.430	10.974
Vitamin A	3.355	1.182
Vitamin B1	6.742	3.619
Vitamin B2	3.511	1.877
Vitamin D	3.987	2.078
Vitamin E	3.432	2.345
Vitamin B9	2.531	5.099
Vitamin K	4.234	2.131
Vitamin B5	3.333	2.200
Vitamin B12	1.010	7.615

**Figure 7:**
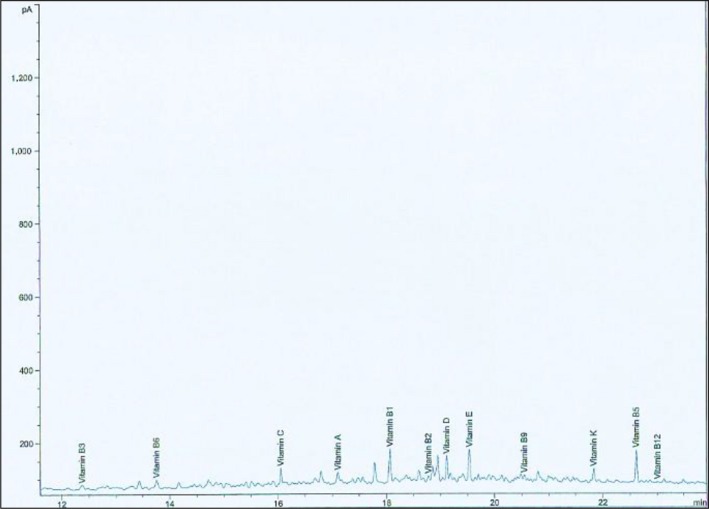
GC‐MS chromatogram of vitamins contained in methanol leaf extract of *Desplatsia dewevrei*

**Figure 8:**
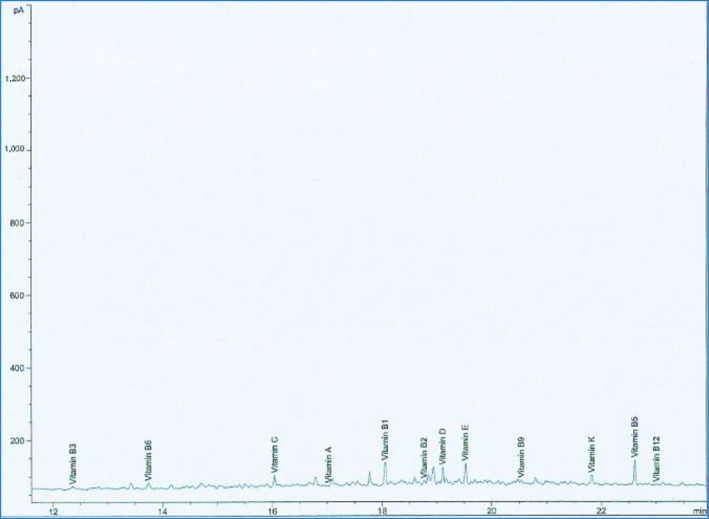
GC‐MS chromatogram of vitamins contained in *Desplatsia dewevrei* fruit extract

The GC‐MS spectra of the vitamins contained in the methanol leaves and fruit extract are shown in Figures 7 and 8, respectively.

## 4 DISCUSSION

According to Okogun (1985), “diets containing an abundance of fruits and vegetables are protective against a variety of diseases, particularly cardiovascular diseases.” In this study, the leaves and fruits of *D. dewevrei* were found to be principally rich in carbohydrate and fiber content (Figures 1 and 2). Foods with high crude fiber values have been implicated in aiding bowel movement because of the cellulose that absorbs water and provides bulk for better functioning of the alimentary system (Wardlaw & Smith, 2009). Fiber may also be functional in food enrichment. Ample intake of dietary fiber had been reported by Ishida, Suzuno, Sugiyama, Innami, and Todokoro (2000) to lower serum cholesterol level, the risk of coronary heart diseases, hypertension, constipation, and diabetes. Fruit fiber is considered to be of better quality than plant fiber. This is maybe due to higher total and soluble fiber content, water and oil holding capacity, and colonic fermentability, as well as lower phytic acid content and caloric value (Anonymous, 2005; Figuerola, Hurtado, Estevez, Chiffelle, & Asenjo, 2005). This suggests that the high fiber content in *D. dewevrei* leaves and fruits may potentiate positive effects in preventing the risk of some colorectal cancers.

The carbohydrate contents in *D. dewevrei* leaves and fruits were also remarkable. Carbohydrate forms a major class of naturally occurring organic compounds which are vital for the upkeep of life in both plants and animals and also offer raw materials for many industries (Ebunoluwa, Adetanwa, Olabisi, & Sina, 2007). This suggests that *D. dewevrei* might be a good source of concentrated dietary energy.

High moisture content promotes susceptibility to microbial activity and spoilage (Ponnusha, Subramaniyan, Pasupathi, Subramaniyan, & Virunandy, 2011). *Desplatsia dewevrei* leaves and fruits may be said to have appreciable moisture content in contrast to that recorded for albedo (Oikeh, Oriakhi, & Omoregie, 2013). However, apt drying of this *D. dewevrei* leaves and fruits is recommended especially when they are harvested for use as herbal remedies to preserve the integrity of the phytochemicals in them and also to prevent microbial attack leading to microbial growth and decay.

Anti‐nutritional plant chemicals like phytates and oxalates though inherently toxic and noncurative in nature have been found to be to play significant roles in the nutritional quality of food (Lewis & Fenwick, 1987; Liener, 1969; Nwokolo & Bragg, 1977). The phytic acid which is the principal store of phosphate is a natural plant oxidant that has shown protective action in carcinogenesis. This action can be explained by its mineral chelating potential. Phytic acid may have health benefits for patients with diabetes as it reduces blood glucose response, by lowering the rate of starch breakdown. Phytic acid also releases inositol during digestion, which might reduce depression. It also reduces inflammation (Idu & Igeleke, 2012). Phytate has been reported to have anticancer properties (Jenab & Thompson, 2000). Phytates are indigestible to humans or nonruminant animals, but may be a source of either inositol or phosphate if eaten directly (Idu, Ovuakporie‐Uvo, & Nwaokolo, 2016). Moreover, they chelate, thus making nonabsorbable certain important minor minerals such as zinc and iron and, to a lesser extent, also macrominerals such as calcium and magnesium (Greiner, Konietzny, & Jany, 2006).

Oxalates are a common constituent of plants. Studies have shown that oxalate can perform different roles in plants, including calcium regulation, ion balance, plant protection, tissue support, and heavy metal detoxification (Idu et al., 2016; Rahman & Kawamura, 2011). Oxalates provide essential nutrients, fiber, antioxidants, and other important phytochemicals. Figures 3 and 4 show that the phytic and oxalic acid contents of *D. dewevrei* are slightly more in the leaves than in the fruits. However, both phytic and oxalic acid contents of *D. dewerei* are together less than those found present in LPC, cooked and sun‐dried *Cnidoscolusa conitifolius* (Aye, 2012), and the FAO permissible daily required intake.

The mineral and elemental composition of *D. dewevrei* shown on Table 1 reveal the presence of selenium, chromium, copper, manganese, iron, sodium, calcium, magnesium, potassium, and zinc in the leaves and fruits at appreciable levels. Copper, calcium, magnesium, and potassium which have been recommended as therapeutically useful are more inherent in leaves than in the fruits of *D. dewevrei*. Copper plays a role in the oxidative defense system but chronic copper toxicity can cause severe poisoning (Uriu‐Adams & Keen, 2005). In this study, the copper concentration in the leaves of *D. dewevrei* was more than in the fruits (Table 1). However, both concentrations of copper were less than the WHO recommended the level of copper in the acceptable range of 20µg/mg body weight per day (FDA, 1993; Watson, 1993). However, copper could be poisonous depending on the dose and duration of exposure (Obi, Akunyili, Ekpo, & Orisakwe, 2006). Copper has been reported to potentiate anti‐inflammatory activities (Rajeshwari & Andallu, 2011). Copper is an essential part of several enzymes and it is necessary to the synthesis of hemoglobin, and its deficiency can lead to anemia and hypoproteinemia (Ahmed, Rehman, Qadiruddin, & Badar, 1994; Idu *et al*., 2015). Calcium, potassium, magnesium, and sodium being macronutrients are important for the proper functioning of vital organs in the body. They also play vital roles as structural and functional components of metalloproteins and enzymes in living cells (Ansari, Ahmad, & Aafaqi, 2004; Zaidi, Asrar, Mansoor, & Farooqui, 2005). Magnesium is a component of chlorophyll and an important mineral element with reference to the prevention of heart diseases and calcium metabolism in bones (Idu, Erhabor, Timothy, & Ovuakporie‐Uvo, 2014; Ishida et al., 2000). It orchestrates the electric current that sparks through the body nerves (Idu & Igeleke, 2012). Magnesium is an anti‐inflammatory and antidiabetic agent (Rajeshwari & Andallu, 2011). Magnesium is a central mineral for retention of vitamin D and calcium into the bones for maintaining bone structure and helps in prevention of osteoporosis and cardiovascular diseases (Chakraborti et al., 2002). According to Hooker (1987), magnesium plays an important role in the metabolism of cholesterol and heart diseases. Sodium present in salt regulates the amount of fluid that the body contains, eliminates fluid waste through urine, and maintains fluid balance (Akpanyung, 2005). Iron is an important constituent of hemoglobin. The leaves of *D. dewevrei* have an appreciable amount of calcium which is good for growth and maintenance of bones, teeth, and muscles (Mohd, Idris, & Abdulrasheed, 2006). Zinc is involved in the normal function of immune system and is a component of over 50 enzymes in the body (Okaka, Akobundu, & Okaka, 2006). The leaves of *D. dewevrei* from literature can supplement the daily requirements of calcium, sodium, magnesium, and zinc.

Essential amino acids also referred to as “indispensable amino acid” are the most important for normal human body physiology and must be supplied to the body through diet. Essential amino acids are nine in total, namely histidine, tryptophan, phenylalanine, leucine, isoleucine, lysine, valine, methionine, and threonine (Banipal, Kaur, Kaur, Gupta, & Banipal, 2015). The synthesis of other amino acids and proteins also requires essential amino acids (Banipal, Kaur, Kaur, Komal, & Banipal, 2016). Essential amino acids are also used to prevent fatigue and improve concentration. Leucine, isoleucine, and valine are imperative for the regulation of carbohydrate metabolism, protein output, and gene expression (Banipal et al., 2015). Peyrollier, Hajduch, Blair, Hyde, and Hundal (2000) reported the key role of leucine as signaling molecule during synthesis of muscle proteins. Additionally, the role of leucine in reversible phosphorylation of ribosomal protein S6 kinase has also been reported for the upregulation of protein synthesis in skeletal muscle (Anthony, Anthony, & Kimball, 2001). The amino acid profile of *D. dewevrei* leaves (5.387 g/100 g) and fruits (4.181 g/100 g) revealed a significant concentration of leucine suggesting that adequate consumption of *D. dewevrei* can help upregulate the protein syntheses of the consumer (Table 2). Valine serves as a “central building block of various enzymes and is vital for supplying energy aiding to muscle cell repair and tissue recovery.” It is also useful in process of beverage fermentation (Anthony et al., 2001). The valine content of the leaves (3.170 g/100 g) and fruits (2.581 g/100 g) of *D. dewevrei* was found to be higher to *D. guineense* (Ayessou et al., 2014; Kumari, Catherina, Katherine, Chew, & Kah‐Guan, 2017). Nonessential amino acids are said to be dispensable because the body can synthesize them readily. According to Sumalantha, Saravana, and Mohama (2010), arginine which was found present in the leaves (8.154 g/100 g) and fruits (1.822 g/100 g) of *D. dewevrei* has been suggested to potentiate aphrodisiac activities. Thus, *D. dewevrei* may be aphrodisiac at certain concentrations. Arginine improves the circulation, strengthens the immune system, and has a positive influence on male libido (Williams, Abumrad, & Barbul, 2002). Research suggests that the amino acid speeds up the rate of the healing of wounds (Lavie, Hafetz, Luboshitzky, & Lavie, 2003), improves the burning of excess fat (Merimee, Lillicrap, & Rabinowitz, 1965), and can be used in weight‐reducing diets. Its role in decreasing cholesterol levels can be ascribed to its function as a biological precursor of nitric oxide (NO). Arginine helps to stimulate and improve hair growth, and arginine helps to reduce insulin resistance and increases glucose tolerance and insulin sensitivity in type 2 diabetes *mellitus* (Piatti et al., 2001; Wu, Meininger, Knabe, Baze, & Rhoads, 2000). The substantial concentration of arginine in *D. dewevrei* may suggest that it may be useful in cholesterol‐lowering activities in man. Glutamate or glutamic acid is in the same amino acid family group as glutamine. Glutamate is a major component of most natural protein foods such as meat, fish, milk, and some vegetables that play an essential role in human metabolism (Filer & Stegink, 1994; Fernstrom, 2000). It helps transmit messages around the brain and also reduce chest pain commonly associated with coronary heart disease (Niswender & Conn, 2010).

Vitamins A, B_1_, B_2_, B_3_, B_5_, B_6_, B_9_, B_12_, C, D, E, and K were found present in *D. dewevrei* leaves and fruits (Table 3, Figures 7 and 8). However, vitamins B_1_ (6.742 g/100 g),B_3_ (5.360 g/100 g), C (53.430 g/100 g), and K (4.234 g/100 g) were the most prominently appreciable vitamins present in *D. dewerei* leaves which were far more than the quantity of each of the vitamins in the fruits of the plant (Table 3). Vitamins B_1_ and B_3_ otherwise known as thiamine and niacin distinctively are water‐soluble vitamins which function as coenzymes needed for energy metabolism. They are also important for keeping the nervous system, digestive system, and skin healthy (Anonymous, 2005). Vitamin C, also referred to as ascorbic acid, also functions as an antioxidant and a coenzyme needed for protein metabolism. Vitamin C is important for healthy functioning of the immune system and healing of cuts, wounds, and gums. It also aids in iron absorption, prevention of cell damage, reduces the risk of certain cancers, heart, and other diseases (Anonymous, 2005, 2014). High intake of vitamin C helps protect low‐density lipoproteins ex vivo against oxidation keeping the immune system in fine fettle. Vitamin C increases the amount of iron absorbed from some foods by the body (Hillstorm, Yacapin‐Ammons, & Lynch, 2003).

Vitamins D and K are fat‐soluble vitamins responsible for an increase in the amount of calcium and phosphorus absorbs, strengthening them and healthier bones and teeth by depositing calcium and phosphorus from foods into the body. Vitamin D guards against infections by strengthening the immune system (Anonymous, 2014). Vitamin K produces proteins that cause a blood clot in case of bleeding and other body proteins for the bones and kidneys (Vermeer, 1990). According to FAO/WHO (2001), the biologic function of vitamin K is to act as a cofactor for a specific carboxylation reaction that transforms selective glutamate residues to gg‐carboxyglutamate (Gla) residues. Phylloquinone is the major circulating form of vitamin K. Since “there is no evidence of even sub‐clinical deficiencies of hemostatic function, a daily intake of 1 µg/kg may still be used as the basis for the recommended nutrient intake (RNI) for vitamin K.” However, in this study, the vitamin K found to be contained in both leaves and fruits of *D. dewevrei* are beyond the RNI standards of the United States and United Kingdom.

In conclusion, *D. dewevrei* is a nutraceutical freely given by nature. It must be harnessed as fast as possible into the human diet to optimize growth and development of especially growing children in (dying as a result of malnutrition) rural and urban settlements of the world.

## CONFLICT OF INTEREST

The authors declare that they do not have any conflict of interest.

## ETHICAL REVIEW

This study does not involve any human or animal testing.

## INFORMED CONSENT

Written informed consent was obtained from all study participants.
